# Public health round-up

**DOI:** 10.2471/BLT.25.011025

**Published:** 2025-10-01

**Persistent water access inequalities**

One in four people globally still lack access to safe drinking water, leaving billions vulnerable to disease and social exclusion. The new report Progress on household drinking water and sanitation 2000–2024: special focus on inequalities, launched at World Water Week 2025 by the World Health Organization and the United Nations Children’s Fund, shows progress remains uneven. Low-income countries, fragile settings, rural areas, children and minority or indigenous groups face the greatest disparities in water, sanitation and hygiene access.

**Figure:**
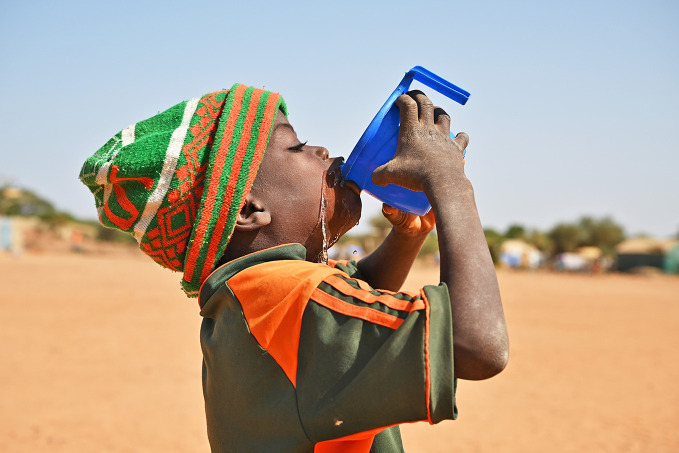
UNICEF/UNI486298/Dejongh

## Tuberculosis research equity

The World Health Organization (WHO) has issued a call to action to end the routine exclusion of pregnant and lactating women from tuberculosis (TB) research. A new consensus statement, developed with over 80 global experts, outlines a framework to ensure equitable access to TB innovations for all, including those most at risk.

Each year, about 200 000 pregnant or postpartum women develop TB, yet they are rarely included in clinical trials. This gap leaves vital questions unanswered and delays access to life-saving treatments and vaccines.

“The benefits of TB research must flow to all people with TB, including pregnant and lactating women,” said Dr Tereza Kasaeva, director of WHO’s Department for HIV, TB, Hepatitis and Sexually Transmitted Infections. “It is time to prioritize their inclusion, not as an afterthought, but as a fundamental step toward equitable, evidence-based care.”

Key actions include closing data gaps, initiating timely preclinical studies, and ensuring inclusion in TB drug and vaccine trials while addressing legal, ethical and regulatory barriers.

https://bit.ly/3Ka1ckb

## Workplace heat stress

WHO and the World Meteorological Organization (WMO) have released new guidance highlighting the growing risks of extreme heat on workers globally. Manual workers in sectors such as agriculture, construction and fisheries are particularly affected, while vulnerable populations in developing countries also face severe health impacts.

“Heat stress is already harming the health and livelihoods of billions of workers, especially in the most vulnerable communities,” said Dr Jeremy Farrar, Assistant Director-General Health Promotion, Disease Prevention and Care. “This new guidance offers practical, evidence-based solutions to protect lives, reduce inequality and build more resilient workforces in a warming world.”

The report, *Climate change and workplace heat stress*, draws on five decades of research, showing that rising temperatures reduce productivity and increase risks of heatstroke, dehydration, kidney dysfunction and neurological disorders. WMO reports that 2024 was the hottest year on record, with daytime temperatures above 40°C becoming increasingly common worldwide.

WHO and WMO recommend occupational heat-health policies, heat action plans tailored to local industries, education for workers and health professionals and stakeholder collaboration. Immediate implementation of these measures is essential to protect the health, safety and economic security of more than 2.4 billion workers exposed to extreme heat globally.

https://bit.ly/3KgwjdP

## Famine confirmed in Gaza 

For the first time, famine has been officially confirmed in the Gaza Strip, where more than half a million people are trapped in extreme hunger, according to a new Integrated Food Security Phase Classification (IPC) analysis. Classifying famine means that the most extreme category is triggered when three critical thresholds, extreme food deprivation, acute malnutrition and starvation-related deaths, have been breached. The latest analysis now affirms that these criteria have been met.

The report warns that currently over 640 000 people are facing catastrophic food insecurity (IPC Phase 5), with more than 1.14 million in emergency condition (IPC Phase 4). Children and pregnant or breastfeeding women are especially affected.

The Food and Agriculture Organization of the United Nations (FAO), the United Nations Children's Fund (UNICEF), the United Nations World Food Programme (WFP) and WHO have collectively and consistently highlighted the extreme urgency for an immediate and full-scale humanitarian response given the escalating hunger-related deaths, rapidly worsening levels of acute malnutrition and plummeting levels of food consumption.

“A ceasefire is an absolute and moral imperative now,” said WHO Director-General Tedros Adhanom Ghebreyesus. “The world has waited too long, watching tragic and unnecessary deaths mount from this man-made famine. Widespread malnutrition means that even common and usually mild diseases like diarrhoea are becoming fatal, especially for children.”

https://bit.ly/46bh2DH

## Tobacco linked to stunting 

WHO has warned that tobacco use contributes to child stunting, a condition affecting nearly 150 million children worldwide. Stunting increases risks of disease, delayed development and death. A new WHO summary highlights evidence linking tobacco exposure during pregnancy and childhood to impaired growth, urging governments to strengthen tobacco control policies.

Children, whose parents smoke, face higher risks of stunting, with stronger effects from maternal smoking during pregnancy. Harmful outcomes include preterm birth, low birth weight and restricted fetal growth, while second-hand smoke after birth worsens infections and development problems. The document stresses that quitting smoking during pregnancy improves growth outcomes.

“Tobacco robs children of their right to grow, learn and thrive,” said Dr Etienne Krug, director of WHO’s Department of Health Determinants, Promotion and Prevention.

WHO calls on countries to fully implement the WHO Framework Convention on Tobacco Control and its MPOWER measures, including enforcing smoke-free public spaces, supporting cessation services and protection for pregnant women and children.

While strong evidence already links tobacco exposure to impaired child growth, WHO calls for further research to deepen understanding of the mechanisms and the benefits of cessation on stunting reduction.

https://bit.ly/4ptAH9z

## Global cholera surge

The recently published *Global cholera statistics 2024* reports an increase in both the number of people who fell sick and who died from the disease. More than 6 000 people died, a 50% increase from 2023, while reported cases grew by 5%. 

Sixty countries reported cholera cases, up from 45 in 2023, with outbreaks concentrated in the African, Eastern Mediterranean and South-East Asia regions. Twelve countries recorded more than 10 000 cases each, including seven experiencing major outbreaks for the first time. In Comoros, cholera returned after 15 years without reported transmission.

Conflict, climate change, population displacement and long-term deficiencies in water, sanitation and hygiene infrastructure continue to drive the rise of cholera, a disease caused by the bacterium, *Vibrio cholerae*, which spreads through faeces-contaminated water.

Preliminary data show that the global cholera crisis is continuing into 2025, with 31 countries reporting outbreaks since the beginning of the year. 

WHO urged stronger surveillance, vaccination, and investment in water and sanitation systems to curb cholera’s spread, warning that the global risk remains very high.

https://bit.ly/48ouTYK

## Essential medicines lists update

On 5 September 2025, WHO released updated editions of its *Model list of essential medicines *(EML) and *Essential medicines for children* (EMLc), adding new treatments for various types of cancer and diabetes with associated comorbidities, such as obesity. Medicines for cystic fibrosis, psoriasis, haemophilia and other blood-related disorders are among the further additions.

“The new editions of essential medicines lists mark a significant step toward expanding access to new medicines with proven clinical benefits and with high potential for global public health impact,” said Dr Yukiko Nakatani, Assistant Director-General for Health Systems, Access and Data.

https://bit.ly/4mjjuNb

## Mental health care gaps

More than one billion people live with mental health conditions, according to new data released by WHO in two major reports, *World mental health today* and *Mental health atlas 2024*. 

Mental health disorders, particularly anxiety and depression, disproportionately affect women and contribute to economic losses of about 1 trillion United States dollars annually. Suicide remains a leading cause of death among young people, yet progress toward global reduction targets is far off track. The findings highlight the urgent need for greater investment, prioritization and collaboration to expand care, reduce stigma and address underlying causes.

The atlas also shows little progress in financing, with mental health spending still at just 2% of health budgets and major workforce shortages persisting. WHO calls on governments to urgently increase investment, reform laws, and expand community-based, rights-focused care to close treatment gaps and protect mental well-being.

“Transforming mental health services is one of the most pressing public health challenges,” said Dr Tedros Adhanom Ghebreyesus, WHO Director-General. “Investing in mental health means investing in people, communities and economies, an investment no country can afford to neglect.”

https://bit.ly/3Ius11W

**Cover photo**

A patient at Al-Mawasi Field Hospital, occupied Palestinian territory, being prepared for medical evacuation, September 2025.

**Figure:**
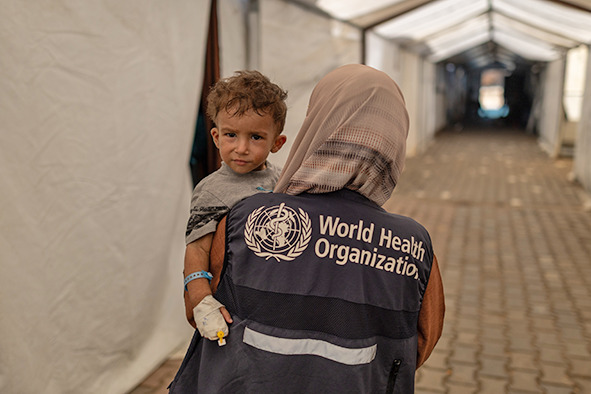
WHO

**Looking ahead**

14–16 October. 28th meeting of the Malaria Policy Advisory Group. https://bit.ly/4gxJKlV

20–22 October. 48th Expert Committee on Drug Dependence. https://bit.ly/46hXM68

28–30 October. Annual general meeting of the WHO Alliance for Food Safety. Muscat, Oman. https://bit.ly/3InxnMu

